# *RACK1* is a candidate gene associated with the prognosis of patients with early stage non-small cell lung cancer

**DOI:** 10.18632/oncotarget.2865

**Published:** 2015-01-09

**Authors:** Yi-Young Choi, Shin Yup Lee, Won Kee Lee, Hyo-Sung Jeon, Eung Bae Lee, Hyun Cheol Lee, Jin Eun Choi, Hyo-Gyoung Kang, Eun Jin Lee, Eun Young Bae, Seung Soo Yoo, Jaehee Lee, Seung Ick Cha, Chang Ho Kim, In-San Kim, Myung Hoon Lee, Young Tae Kim, Sanghoon Jheon, Jae Yong Park

**Affiliations:** ^1^ Departments of Biochemistry and Cell Biology, Kyungpook National University, Daegu, Republic of Korea; ^2^ Internal Medicine, School of Medicine, Kyungpook National University, Daegu, Republic of Korea; ^3^ Lung Cancer Center, Kyungpook National University Medical Center, Daegu, Republic of Korea; ^4^ Biostatistics Center, School of Medicine, Kyungpook National University, Daegu, Republic of Korea; ^5^ Department of Thoracic Surgery, School of Medicine, Kyungpook National University, Daegu, Republic of Korea; ^6^ Diagnosis and Prediction Biotechnology, School of Medicine, Kyungpook National University, Daegu, Republic of Korea; ^7^ Department of Thoracic and Cardiovascular Surgery, Seoul National University School of Medicine, Seoul, Republic of Korea

**Keywords:** polymorphism, RACK1, lung cancer, survival

## Abstract

**Background:**

This study was conducted to identify genetic polymorphisms associated with the prognosis of patients with early stage NSCLC.

**Materials and Methods:**

We genotyped 1,969 potentially functional single nucleotide polymorphisms (SNPs) of 1,151 genes involved in carcinogenesis in 166 NSCLC patients who underwent curative surgery, using the Affymetrix custom-made GeneChip. A replication study was performed in an independent cohort of 626 patients.

**Results:**

Fifty six SNPs which were associated with both overall survival (OS) and disease-free survival (DFS) with log-rank *P* values < 0.05 in discovery set were selected for validation. Among those, five SNPs (*RACK1* rs1279736C>A and rs3756585T>G, *C3* rs2287845T>C, *PCAF* rs17006625A>G, and *PCM1* rs17691523C>G) were found to be significantly associated with survival in the same direction as the discovery set. In combined analysis, the rs1279736C>A and rs3756585T>G were most significantly associated with OS and DFS in multivariate analysis (*P* for OS = 4 × 10^−5^ and 7 × 10^−5^, respectively; and *P* for DFS = 0.003, both; under codominant model). *In vitro* promoter assay and electrophoretic mobility shift assay revealed that the rs3756585 T-to-G change increased promoter activity and transcription factor binding of *RACK1*.

**Conclusions:**

We identified five SNPs, especially *RACK1* rs3756585T>G, as markers for prognosis of patients with surgically resected NSCLC.

## INTRODUCTION

Lung cancer, predominantly non-small cell lung cancer (NSCLC), is the leading cause of cancer-related deaths worldwide, with an average 5-year survival rate of 15% [1]. Surgery is the best treatment modality for potential cure in early stage NSCLC, but a large proportion of the patients ultimately die from disease recurrence. The 5-year survival rates range from 73% for stage IA to 36% for stage II [2]. It is thus likely that many cancers diagnosed at early stage have already spread at microscopic level and tumors vary in their biologic behavior. Although pathologic tumor stage is the most important predictor of prognosis after surgical resection, patients with the same pathologic stage display marked variability in recurrence and survival [2]. Therefore, intensive research is currently in progress for prognostic biomarkers that would allow more precise identification of patients with the highest or lowest risk of relapse following surgery [3]. Given that adjuvant therapies with efficacy in some patients are now available [4, 5], the biomarkers to predict recurrence and prognosis after lung cancer surgery is even more important because they could help to select subgroups of patients for adjuvant treatment, either with conventional cytotoxic chemotherapy or novel targeted therapeutic agents.

Carcinogenesis is a multi-step process characterized by the accumulation of multiple genetic and epigenetic alterations, which results in alterations in cell physiology that collectively dictate malignant growth: self-sufficiency of growth signals, insensitivity to growth-inhibitory signals, evasion of apoptosis, limitless replicative potential, sustained angiogenesis, and tissue invasion and metastasis [6]. Although many genes not previously suspected of having a role in lung carcinogenesis may contribute to the prognosis of lung cancer, we hypothesized that genetic variants in genes known to play important roles in the development and progression of cancer may influence the prognosis of lung cancer. To test this hypothesis, we evaluated the associations between potentially functional variants in cancer-related genes and prognosis of lung cancer.

## RESULTS

### Patient Characteristics and Clinical Predictors

The clinical and pathologic characteristics of the patients in discovery and validation sets and the association with OS and DFS are shown in Table 1. There is no difference in distribution of age, sex, pack-years in smokers, and pathologic stage between the discovery and validation sets. However, never smokers and adenocarcinomas are significantly more frequent in the validation set compared with the discovery set (*P* = 0.01 and 0.001, respectively). Upon univariate analysis, pathologic stage was significantly associated with OS and DFS (both, log-rank *P* [*P_L–R_*] ≤ 0.0001) in both sets. Age was associated with OS and DFS in the validation set (*P_L–R_* for OS = 0.003 and *P_L–R_* for DFS = 0.03), and gender and smoking status were also associated with OS in the validation set (*P_L–R_* for OS = 0.02 and 0.01, respectively).

**Table 1:** Univariate analysis for overall survival and disease-free survival by clinicopathologic features of the discovery and validation cohorts

	Discovery set							Validation set						
		Overall Survival			Disease-Free Survival				Overall Survival			Disease-Free Survival		
Variables	No. of patients(%)*	No. of deaths(%)^†^	5Y-OSR (%)^‡^	Log-Rank *P*	No. of events(%)^†^	5Y-DFSR (%)^‡^	Log-Rank *P*	No. of patients(%)*	No. of deaths(%)^†^	5Y-OSR (%)^‡^	Log-Rank *P*	No. of events(%)^†^	5Y-DFSR (%)^‡^	Log-Rank *P*
Overall	166	40 (24.1)	65		64 (38.6)	53		626	182(29.1)	67		306(48.9)	47	
Age, years														
≤ 63	84 (50.6)	16 (19.1)	73	0.08	32 (38.1)	59	0.68	338 (54.0)	86(25.4)	73	**0.003**	156(46.2)	52	**0.03**
> 63	82 (49.4)	24 (29.3)	56		32 (39.0)	47		288 (46.0)	96(33.3)	59		150(52.1)	41	
Sex														
Male	127 (76.5)	32 (25.2)	65	0.69	47 (37.0)	54	0.35	449 (71.7)	142(31.6)	64	**0.02**	222(49.4)	47	0.67
Female	39 (23.5)	8 (20.5)	65		17 (43.6)	48		177 (28.3)	40(22.6)	74		84(47.5)	47	
Smoking status														
Never	38 (22.9)	9 (23.7)	66	0.99	17 (44.7)	47	0.37	207 (33.1)^§^	49(23.7)	73	**0.01**	101(48.8)	45	0.65
Ever	128 (77.1)	31 (24.2)	65		47 (36.7)	54		419 (66.9)	133(31.7)	64		205(48.9)	48	
Pack-years^#^														
≤ 40	85 (66.4)	19 (22.4)	69	0.29	31 (36.5)	56	0.92	255 (60.9)	77(30.2)	65	0.38	122(47.8)	48	0.63
> 40	43 (33.6)	12 (27.9)	54		16 (37.2)	52		164 (39.1)	56(34.2)	62		83(50.6)	49	
Histological types														
SCC	96 (57.8)	22 (22.9)	68	0.84	35 (36.5)	54	0.68	264 (42.2)^¶^	77(29.2)	67	0.39	113(42.8)	55	0.09
AC	66 (39.8)	17 (25.8)	60		28 (42.4)	49		338 (54.0)	95(28.1)	67		178(52.7)	42	
LCC	4 (0.02)	1 (25.0)	75		1 (25.0)	75		24 (3.8)	10(41.7)	60		15(62.5)	46	
Pathologic stage														
I	97 (58.4)	13 (13.4)	78	**0.001**	22 (22.7)	69	**2 × 10^−6^**	381 (60.9)	89(23.4)	71	**4 × 10^−5^**	152(39.9)	54	**1 × 10^−9^**
II+IIIA	69 (41.6)	27 (39.1)	49		42 (60.9)	32		245 (39.1)	93(38.0)	61		154(62.9)	36	

Abbreviations: OSR, overall survival rate; DFSR, disease-free survival rate; SCC, squamous cell carcinoma; AC, adenocarcinoma; LCC, large cell carcinoma

* Column percentage

† Row percentage

‡ Five year-overall survival rate (OSR) and 5 year-disease free survival rate (DFSR), proportion of survival derived from Kaplan-Meier analysis.

# In ever-smokers.

§ *P* = 0.01

¶ *P* = 0.001

### Associations between SNPs and survival outcomes

From the 1,969 SNPs genotyped, we excluded (i) 81 SNPs with genotype failure, (ii) 166 with genotype call rate < 90%, (iii) 211 with minor allele frequency < 5%, or (iv) 126 showing deviation from Hardy-Weinberg equilibrium (*P* < 0.05), thus analyzed 1,385 SNPs in 910 genes. Approximately 49% of the SNPs were located in promoter region, 23% in exons (nonsynonymous SNPs), 15% in exon-intron boundaries, 7% in 5′-UTRs, and 6% in 3′-UTRs.

**Figure 1:**
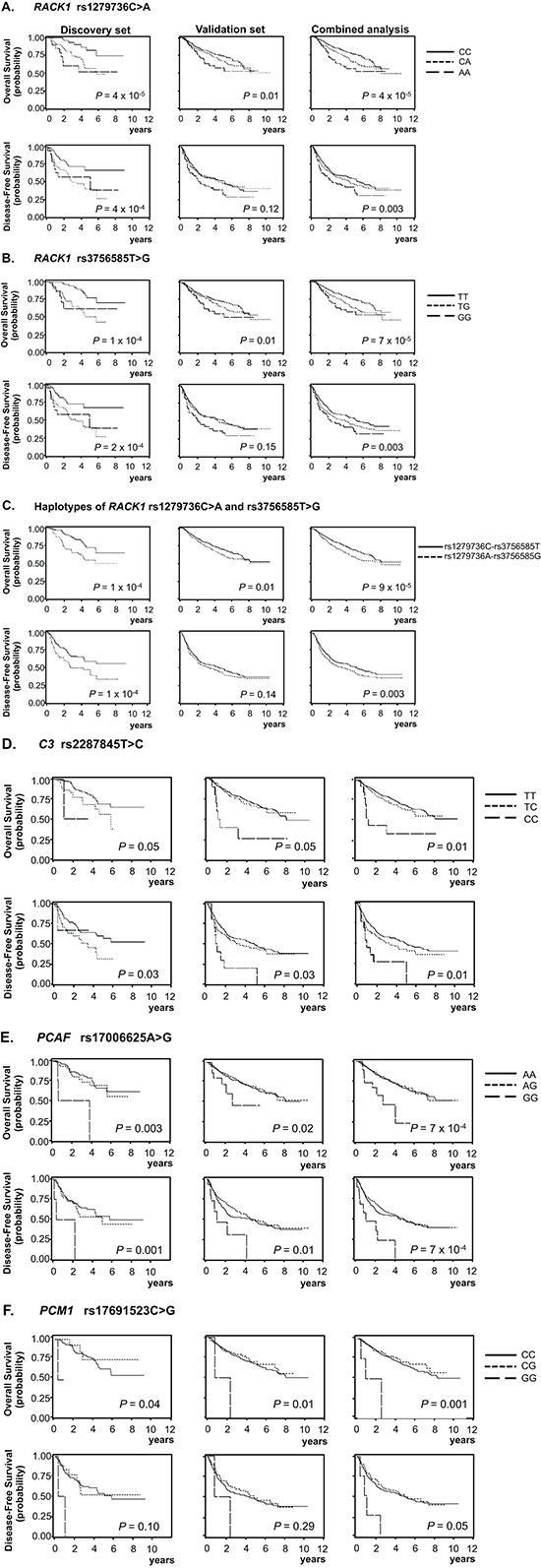
Kaplan-Meier plots of overall survival and disease-free survival according to genotypes and haplotypes *RACK1* rs1279736C>A, **(A)**; *RACK1* rs3756585T>G, **(B)**; Haplotypes of *RACK1* rs1279736C>A and rs3756585T>G, **(C)**; *C3* rs2287845T>C, **(D)**; *PCAF* rs17006625A>G, **(E)**; and *PCM1* rs17691523C>G, **(F)**; *P* values in the multivariate Cox proportional hazard model; codominant model for A, B, and D, and recessive model for E, and F.

Of the 1,385 SNPs analyzed in the discovery set, 56 SNPs were associated with both OS and DFS with *P_L-R_* < 0.05, and selected for validation (Table 2). Among the 56 SNPs, five SNPs (receptor for activated C kinase 1 [*RACK1*] rs1279736C>A and rs3756585T>G, complement component 3 [*C3*] rs2287845T>C, p300/CBP-associated factor [*PCAF*] rs17006625A>G, and pericentriolar material 1 [*PCM1*] rs17691523C>G) were found to be significantly associated with survival outcomes in the same direction as the discovery set in an independent validation set (Table 3 and Figure 1A–1F) when adjusted for age, gender, smoking status, tumor histology, and pathologic stage. For each of the five SNP, the survival figures of each stage showed similar pattern compared with those including patients of all stages (Supplementary Figure 1A–1E). In combined analysis, the most significant two SNPs (rs1279736 and rs3756585) were located in the promoter of *RACK1* gene (−283 and −123 from transcription start site, respectively). Adjusted HRs (aHRs) for OS of the rs1279736 and rs3756585 were 1.57 and 1.54, respectively (*P* = 4 × 10^−5^ and 7 × 10^−5^, respectively) and aHRs for DFS of the two SNPs were 1.28, both (*P* = 0.003, both), under a codominant model for the variant allele at each loci. The two SNPs were in strong linkage disequilibrium (LD) (|D’| = 1.0 and *r*^2^ = 0.91) with two predominant haplotypes accounting for more than 98% of the haplotypes in the subjects. Consistent with the results of genotyping analyses, the rs1279736A-rs3756585G haplotype carrying variant alleles at both loci was associated with significantly worse survival outcomes compared to the rs1279736C-rs3756585T haplotype carrying wild-type alleles at both loci (aHR for OS = 1.48, 95% CI = 1.22–1.80, *P* = 9 × 10^−5^, and aHR for DFS = 1.26, 95% CI = 1.08–1.47, *P* = 0.003).

**Table 2:** Summary of SNPs analyzed in the discovery and validation studies

	Discovery (*n* = 166)							Validation (*n* = 626)						
ID No.	MAF	P^*^ for OS			P^*^ for DFS			MAF	P^*^ for OS			P^*^ for DFS		
		Dominant	Recessive	Codominant	Dominant	Recessive	Codominant		Dominant	Recessive	Codominant	Dominant	Recessive	Codominant
rs1279736C>A	0.30	**0.001**	**0.03**	**0.0008**	**0.003**	0.43	**0.01**	0.31	0.12	0.07	0.16	0.55	**0.03**	0.16
rs3756585T>G	0.34	**0.0002**	0.29	**0.001**	**0.0002**	0.97	**0.006**	0.32	0.09	0.07	**0.03**	0.41	**0.03**	0.12
rs2287845T>C	0.15	**0.03**	0.28	**0.03**	**0.02**	0.84	**0.03**	0.13	0.32	**0.0004**	0.08	0.09	**0.004**	**0.03**
rs2232946C>T	0.47	**0.02**	0.02	**0.005**	**0.03**	0.17	**0.03**	0.40	0.13	0.30	0.12	0.92	0.72	0.90
rs17691523C>G	0.07	0.66	**< 0.0001**	0.98	0.54	**0.001**	0.23	0.07	0.95	**0.001**	0.76	0.66	0.15	0.83
rs4280262A>G	0.10	0.22	**<0.0001**	0.11	**0.004**	**0.02**	**0.002**	0.09	0.55	0.92	0.56	0.53	0.37	0.44
rs11868429C>T	0.09	0.58	**<0.0001**	0.34	0.63	**0.03**	0.85	0.10	0.37	0.72	0.37	0.71	0.90	0.72
rs11168070C>G	0.09	0.41	**0.002**	0.15	0.21	**<0.0001**	0.07	0.09	0.55	0.53	0.49	0.14	0.88	0.19
rs5447A>G	0.10	0.63	**0.04**	0.41	0.75	**<0.0001**	0.52	0.11	0.60	0.96	0.65	0.47	0.70	0.59
rs10416620C>G	0.12	0.46	**0.009**	0.97	0.42	**0.02**	0.76	0.11	0.13	**0.01**	**0.05**	0.39	0.08	0.25
rs4745A>T	0.14	0.11	**0.01**	0.34	0.56	**0.0007**	0.94	0.13	0.43	0.55	0.39	0.75	0.91	0.74
rs10138227C>T	0.14	**0.02**	0.40	**0.02**	**0.01**	0.97	**0.02**	0.13	0.86	0.37	0.98	0.54	0.22	0.76
rs332271A>G	0.15	0.60	**0.0006**	0.20	0.53	**0.01**	1.00	0.14	0.40	0.77	0.46	0.85	0.34	0.96
rs1863494A>T	0.16	0.66	**0.0003**	0.32	0.56	**0.01**	0.88	0.15	0.70	0.88	0.76	0.24	0.35	0.19
rs1407267G>T	0.13	0.42	**0.005**	0.85	0.56	**0.01**	0.95	0.16	0.70	0.23	0.95	0.46	0.32	0.74
rs17006625A>G	0.20	0.31	**0.0004**	0.08	0.29	**0.01**	0.12	0.19	0.90	**0.04**	0.55	0.50	**0.01**	0.99
rs10827493C>T	0.24	0.50	**0.001**	0.17	0.33	**0.02**	0.13	0.23	0.29	0.59	0.52	0.21	0.20	0.58
rs708244C>T	0.25	0.12	**0.05**	**0.05**	0.21	**0.01**	**0.04**	0.26	0.74	0.83	0.87	0.98	0.72	0.89
rs1047266C>T	0.24	0.10	**0.04**	**0.04**	0.26	**0.01**	0.09	0.26	0.97	**0.04**	0.47	0.43	**0.01**	0.11
rs12443101A>C	0.31	0.24	**0.03**	0.07	0.64	**0.03**	0.18	0.31	0.49	0.39	0.91	0.27	0.17	0.86
rs11353C>T	0.41	**0.03**	0.50	0.28	**0.01**	0.59	0.15	0.34	0.50	0.52	0.42	0.24	0.94	0.37
rs592121A>G	0.35	0.01	0.53	0.16	**0.03**	0.93	0.14	0.39	0.30	0.15	0.98	0.57	0.92	0.66
rs9722C>T	0.35	**0.04**	0.97	0.10	**0.02**	0.79	**0.05**	0.37	**0.04**	0.10	**0.02**	0.17	**0.03**	**0.04**
rs3200254C>T	0.50	**0.02**	0.81	0.17	**0.02**	0.47	0.31	0.48	0.65	0.67	0.59	0.69	0.80	0.69
rs17110192G>C	0.05	0.88	**<0.0001**	0.71	0.54	**<0.0001**	0.82	0.05	0.07		0.08	**0.05**		**0.05**
rs269868T>C	0.08	0.32	**<0.0001**	0.56	0.82	**<0.0001**	0.56	0.08	0.26	0.80	0.26	0.29	0.50	0.40
rs11959820C>A	0.10	0.09	**0.0002**	**0.05**	0.09	**0.01**	0.06	0.10	0.09	0.36	0.08	0.62	0.23	0.88
rs17499824T>C	0.08	0.76	**<0.0001**	0.45	0.78	**0.003**	0.56	0.10	0.87	0.77	0.95	0.35	0.59	0.49
rs6743139G>A	0.11	0.97	**<0.0001**	0.69	0.93	**<0.0001**	0.70	0.12	0.45	0.85	0.46	0.99	0.94	0.97
rs3756074G>C	0.08	0.12	**0.04**	0.06	0.40	**0.01**	0.26	0.13	0.29	0.34	0.52	0.79	0.07	0.76
rs4148727T>C	0.11	0.03	0.94	**0.03**	**0.01**	0.78	**0.01**	0.12	0.36	0.56	0.32	0.47	0.73	0.45
rs9297G>A	0.14	0.12	**0.01**	0.35	0.58	**0.0007**	0.96	0.12	0.27	0.66	0.26	0.37	0.81	0.43
rs1133328C>A	0.12	0.31	**<0.0001**	**0.05**	0.73	**<0.0001**	0.27	0.14	0.97	0.63	0.91	0.52	0.29	0.79
rs3746266T>C	0.22	0.56	**<0.0001**	0.17	0.48	**0.01**	0.93	0.18	0.40	0.44	0.63	0.59	0.81	0.59
rs3815331T>C	0.25	0.32	**0.05**	0.12	**0.03**	**0.02**	**0.01**	0.19	0.62	0.88	0.64	0.53	0.55	0.77
rs3219463G>A	0.27	0.13	**0.0005**	**0.01**	0.51	**0.01**	0.11	0.29	0.62	0.15	0.31	0.78	0.25	0.47
rs907187G>C	0.48	0.09	**0.03**	**0.02**	0.30	**0.01**	0.02	0.45	0.71	0.56	0.93	0.81	0.89	0.94
rs2075164C>T	0.09	0.01		**0.02**	**0.02**		0.02	0.10	0.58	0.13	0.83	0.96	0.50	0.85
rs1975285C>G	0.14	0.01	0.63	**0.01**	**0.01**	0.47	0.01	0.14	0.87	0.56	0.76	0.49	0.65	0.45
rs12923640A>G	0.20	0.01	0.61	**0.02**	**0.002**	0.55	0.01	0.18	0.38	0.13	0.87	0.54	0.10	0.90
rs3741936C>T	0.32	0.05	**<0.0001**	**0.0009**	0.09	**0.03**	0.02	0.28	0.40	0.18	0.22	0.77	0.64	0.67
rs1336665C>G	0.22	0.73	**0.0002**	0.13	0.32	**0.01**	0.09	0.24	0.65	0.86	0.76	0.70	0.73	0.65
rs6651394C>T	0.44	0.47	**0.003**	**0.03**	0.93	**0.05**	0.25	0.42	0.87	0.71	0.75	0.50	0.34	0.95
rs3181259C>T	0.42	0.44	**0.01**	**0.05**	0.26	**0.01**	0.04	0.44	**0.04**	0.50	0.08	0.10	0.17	0.06
rs2298876A>G	0.36	4 × 10^−4^	0.08	**0.0007**	**0.02**	**0.03**	0.01	0.44	0.09	0.68	0.16	0.08	0.66	0.15
rs3732487A>C	0.47	0.004	0.40	**0.02**	**0.04**	0.21	0.04	0.49	0.27	**0.02**	**0.04**	0.12	0.09	**0.05**
rs2291049C>T	0.49	0.01	0.18	0.40	**0.05**	0.10	0.80	0.46	0.42	0.55	0.39	0.38	0.38	0.28
rs1078979A>G	0.37	0.77	**0.01**	0.30	0.67	**0.01**	0.12	0.36	0.88	0.93	0.88	0.76	0.39	0.52
rs3738952A>G	0.33	0.71	**0.02**	0.45	0.65	**0.02**	0.55	0.30	0.55	0.14	0.26	0.30	0.18	0.16
rs12621138A>T	0.15	0.01	0.78	**0.02**	**0.03**	0.77	0.07	0.13	0.29	0.79	0.31	0.40	0.16	0.24
rs2274976A>G	0.08	0.06	0.10	**0.04**	0.06	**0.0002**	**0.03**	0.08	0.88	0.78	0.83	0.15	0.94	0.20
rs2779248C>T	0.12	0.01	0.07	**0.005**	**0.03**	0.28	**0.03**	0.10	0.68	0.25	0.88	0.91	0.40	0.95
rs580800C>T	0.09	0.02	0.51	**0.03**	**0.01**	0.42	**0.02**	0.06	0.39	0.18	0.28	0.62	0.42	0.74
rs1571767A>T	0.08	0.003	0.09	**0.002**	**0.0005**	**0.0002**	**0.0003**	0.06	0.84	0.61	0.89	0.40	**<0.0001**	0.51

Abbreviations: ID, identification number; MAF, minor allele frequency; OS, overall survival; DFS, disease-free survival.

* Log-rank *P*

**Table 3:** Association of three significant SNPs and survival outcomes in the discovery and validation set

		Discovery set				Validation set				Combined analysis			
Gene/ID No.*	Genotypes*	Overall Survival		Disease-Free Survival		Overall Survival		Disease-Free Survival		Overall Survival		Disease-Free Survival	
		HR (95%CI)^†^	*P*^†^	HR (95%CI)^†^	*P*^†^	HR (95%CI)^†^	*P*^†^	HR (95%CI)^†^	*P*^†^	HR (95%CI)^†^	*P*^†^	HR (95%CI)*	*P*^†^
*RACK1* rs1279736													
	CC	1.00		1.00		1.00		1.00		1.00		1.00	
	CA	4.27(1.73–10.52)	**0.002**	3.01(1.66–5.45)	**3 × 10^−4^**	1.28(0.91–1.79)	0.15	1.01(0.78–1.29)	0.96	1.53(1.12–2.08)	**0.007**	1.21(0.97–1.53)	0.10
			*0.006*		*0.002*		*0.23*		*0.96*		*0.01*		*0.13*
	AA	9.25(3.01–28.46)	**1 × 10^−4^**	3.37(1.41–8.05)	**0.006**	2.01(1.22–3.33)	**0.007**	1.55(1.05–2.27)	0.03	2.49(1.59–3.88)	**6 × 10^−5^**	1.72(1.21–2.44)	**0.002**
			*8 × 10^−4^*		*0.02*		*0.03*		*0.12*		*6 × 10^−4^*		*0.02*
	Dominant	4.86(2.02–11.72)	**4 × 10^−4^**	3.06(1.71–5.48)	**2 × 10^−4^**	1.38(1.00–1.90)	0.05	1.08(0.85–1.38)	0.51	1.66(1.24–2.23)	**8 × 10^−4^**	1.29(1.04–1.60)	0.02
			*0.002*		*0.001*		*0.09*		*0.75*		*0.003*		*0.05*
	Recessive	3.46(1.45–8.26)	**0.005**	1.69(0.79–3.59)	0.17	1.75(1.10–2.79)	**0.02**	1.54(1.07–2.21)	0.02	1.94(1.30–2.89)	**0.001**	1.55(1.12–2.14)	**0.008**
			*0.01*		*0.24*		*0.05*		*0.12*		*0.003*		*0.03*
	Codominant	3.03(1.79–5.13)	**4 × 10^−5^**	2.00(1.37–2.94)	**4 × 10^−4^**	1.38(1.08–1.75)	**0.009**	1.16(0.96–1.39)	0.12	1.57(1.26–1.94)	**4 × 10^−5^**	1.28(1.09–1.51)	**0.003**
			*8 × 10^−4^*		*0.002*		*0.034*		*0.27*		*6 × 10^−4^*		*0.02*
*RACK1* rs3756585													
	TT	1.00		1.00		1.00		1.00		1.00		1.00	
	TG	4.11(1.82–9.28)	**7 × 10^−4^**	3.41(1.92–6.06)	**3 × 10^−5^**	1.29(0.93–1.80)	0.13	1.01(0.78–1.29)	0.96	1.61(1.19–2.18)	**0.002**	1.28(1.02–1.61)	0.03
			*0.003*		*4 × 10^−4^*		*0.21*		*0.96*		*0.004*		*0.06*
	GG	6.39(2.00–20.44)	**0.002**	2.83(1.10–7.31)	0.03	1.96(1.18–3.26)	**0.01**	1.51(1.02–2.24)	0.04	2.28(1.43–3.61)	**5 × 10^−4^**	1.65(1.15–2.37)	**0.007**
			*0.006*		*0.06*		*0.03*		*0.12*		*0.003*		*0.02*
	Dominant	4.42(2.01–9.74)	**2 × 10^−4^**	3.31(1.89–5.78)	**3 × 10^−5^**	1.38(1.01–1.90)	0.05	1.08(0.85–1.37)	0.55	1.70 (1.27–2.28)	**4 × 10^−4^**	1.34(1.07–1.66)	**0.009**
			*0.001*		*4 × 10^−4^*		*0.09*		*0.76*		*0.003*		*0.03*
	Recessive	2.96(1.05–8.32)	0.04	1.53(0.63–3.70)	0.34	1.70(1.06–2.73)	0.03	1.51(1.04–2.18)	0.03	1.74(1.14–2.66)	**0.01**	1.44(1.03–2.03)	0.03
			*0.08*		*0.41*		*0.06*		*0.12*		*0.02*		*0.06*
	Codominant	2.76(1.65–4.60)	**1 × 10^−4^**	2.04(1.41–2.96)	**2 × 10^−4^**	1.37(1.08–1.73)	**0.01**	1.15(0.95–1.38)	0.15	1.54(1.24–1.90)	**7 × 10^−5^**	1.28(1.09–1.51)	**0.003**
			*8 × 10^−4^*		*0.001*		*0.03*		*0.30*		*6 × 10^−4^*		*0.02*
Haplotypes of *RACK1* rs1279736 and rs3756585													
	ht1. CT	1.00		1.00		1.00		1.00		1.00		1.00	
	ht2. CG	-		-		1.34(0.19–9.78)	0.77	0.67(0.09–4.83)	0.69	1.15(0.16–8.31)	0.89	0.67(0.09–4.79)	0.69
	ht3. AT	2.77(1.20–6.37)	0.02	1.40(0.67–2.96)	0.37	1.27(0.40–3.99)	0.68	0.92(0.34–2.48)	0.87	1.48(0.78–2.80)	0.23	0.94(0.53–1.67)	0.83
	ht4. AG	2.61(1.61–4.23)	1 × 10^−4^	2.09(1.43–3.05)	1 × 10^−4^	1.31(1.06–1.63)	0.01	1.13(0.96–1.34)	0.14	1.48(1.22–1.80)	9 × 10^−5^	1.26(1.08–1.47)	0.003
*C3* rs2287845													
	TT	1.00		1.00		1.00		1.00		1.00		1.00	
	TC	1.90(0.91–3.98)	0.09	2.00(1.13–3.54)	**0.02**	1.13(0.80–1.61)	0.49	1.21(0.93–1.58)	0.16	1.24(0.91–1.68)	0.17	1.28(1.01–1.62)	0.04
			*0.13*		*0.04*		*0.60*		*0.30*		*0.23*		*0.06*
	CC	3.24(0.41–25.37)	0.26	1.25(0.17–9.40)	0.83	3.16(1.46–6.86)	**0.004**	2.08(1.06–4.11)	0.03	3.10(1.51–6.38)	**0.002**	1.99(1.05–3.77)	0.04
			*0.35*		*0.86*		*0.03*		*0.12*		*0.004*		*0.06*
	Dominant	1.97 (0.96–4.05)	0.06	1.94(1.11–3.39)	**0.02**	1.24(0.89–1.73)	0.20	1.27(0.98–1.63)	0.07	1.34(1.00–1.79)	0.05	1.33(1.06–1.67)	**0.01**
			*0.10*		*0.04*		*0.27*		*0.17*		*0.07*		*0.04*
	Recessive	2.76(0.36–21.34)	0.33	1.06(0.14–7.87)	0.96	3.07(1.42–6.62)	**0.004**	1.98(1.01–3.89)	0.05	2.94(1.44–6.02)	**0.003**	1.87(0.99–3.53)	0.05
			*0.41*		*0.96*		*0.03*		*0.13*		*0.006*		*0.08*
	Codominant	1.87 (1.00–3.49)	0.05	1.67(1.04–2.68)	0.03	1.33(1.00–1.78)	0.05	1.28(1.03–1.61)	0.03	1.40(1.08–1.81)	**0.01**	1.32(1.08–1.62)	**0.006**
			*0.08*		*0.06*		*0.09*		*0.12*		*0.02*		*0.02*
*PCAF* rs17006625													
	AA	1.00		1.00		1.00		1.00		1.00		1.00	
	AG	1.08(0.53–2.18)	0.84	1.21(0.70–2.07)	0.50	0.96(0.69–1.33)	0.80	0.88(0.68–1.13)	0.31	0.99(0.74–1.33)	0.93	0.93(0.74–1.17)	0.52
			*0.87*		*0.57*		*0.84*		*0.48*		*0.97*		*0.60*
	GG	8.68(2.1–35.9)	**0.003**	11.03(2.70–45.15)	**8 × 10^−4^**	2.57(1.11–5.97)	0.03	2.3(1.20–4.41)	0.01	3.22(1.62–6.41)	**9 × 10^−4^**	2.6(1.47–4.59)	**0.001**
			*0.007*		*0.003*		*0.06*		*0.12*		*0.003*		*0.01*
	Dominant	1.32(0.68–2.55)	0.41	1.36(0.81–2.28)	0.25	1.02(0.74–1.4)	0.90	0.93(0.73–1.19)	0.58	1.07(0.81–1.42)	0.63	0.99(0.80–1.24)	0.95
			*0.49*		*0.33*		*0.90*		*0.76*		*0.69*		*0.98*
	Recessive	8.49(2.09–34.52)	**0.003**	10.35(2.58–41.63)	**0.001**	2.61(1.14–6.01)	0.02	2.41(1.26–4.59)	0.008	3.24(1.64–6.39)	**7 × 10^−4^**	2.67(1.52–4.69)	**7 × 10^−4^**
			*0.007*		*0.003*		*0.06*		*0.12*		*0.003*		*0.01*
	Codominant	1.59(0.88–2.85)	0.12	1.55(0.95–2.51)	0.08	1.1(0.83–1.48)	0.51	1.02(0.81–1.27)	0.90	1.18(0.91–1.53)	0.20	1.08(0.88–1.33)	0.44
			*0.17*		*0.14*		*0.60*		*0.96*		*0.25*		*0.55*
*PCM1* rs17691523													
	CC	1.00		1.00		1.00		1.00		1.00		1.00	
	CG	0.77(0.23–2.57)	0.67	1.26(0.55–2.86)	0.59	1.06(0.67–1.66)	0.81	0.96(0.68–1.36)	0.83	1.00(0.66–1.53)	0.99	1.0(0.73–1.38)	0.98
			*0.73*		*0.64*		*0.84*		*0.96*		*0.99*		*0.98*
	GG	10.36(1.05–102.28)	0.05	3.88(0.81–8.53)	0.09	6.6(1.59–27.32)	**0.009**	2.12(0.52–8.66)	0.29	6.94(2.16–2.33)	**0.001**	2.71(1.00–7.38)	0.05
			*0.08*		*0.15*		*0.03*		*0.48*		*0.003*		*0.07*
	Dominant	0.99(0.34–2.89)	0.99	1.47(0.70–3.09)	0.31	1.14(0.74–1.75)	0.56	0.99(0.71–1.39)	0.95	1.11(0.74–1.65)	0.62	1.06(0.78–1.44)	0.71
			*0.99*		*0.39*		*0.64*		*0.96*		*0.69*		*0.77*
	Recessive	10.75(1.09–106.01)	0.04	3.75(0.79–7.74)	0.10	6.55(1.58–27.1)	**0.01**	2.13(0.52–8.69)	0.29	6.94(2.16–22.3)	**0.001**	2.71(1.00–7.37)	0.05
			*0.08*		*0.15*		*0.03*		*0.47*		*0.003*		*0.07*
	Codominant	1.23(0.48–3.14)	0.66	1.55(0.84–2.86)	0.16	1.22(0.81–1.84)	0.34	1.02(0.74–1.41)	0.91	1.21(0.83–1.76)	0.32	1.11(0.84–1.48)	0.46
			*0.73*		*0.24*		*0.45*		*0.96*		*0.38*		*0.55*

* Information about polymorphisms and IDs were obtained from NCBI database (http://www.ncbi.nlm.nih.gov/SNP). In the reference sequence, the transcription start site was counted as +1.

† HRs, 95% CIs and their corresponding *P*-values were calculated using multivariate Cox proportional hazard models, adjusted for age, gender, smoking status, tumor histology, pathologic stage; *P*-values in italics represent corrected *P*-values by FDR.

### The effect of rs1279736C>A and rs3756585T>G on the promoter activity of *RACK1*

**Figure 2:**
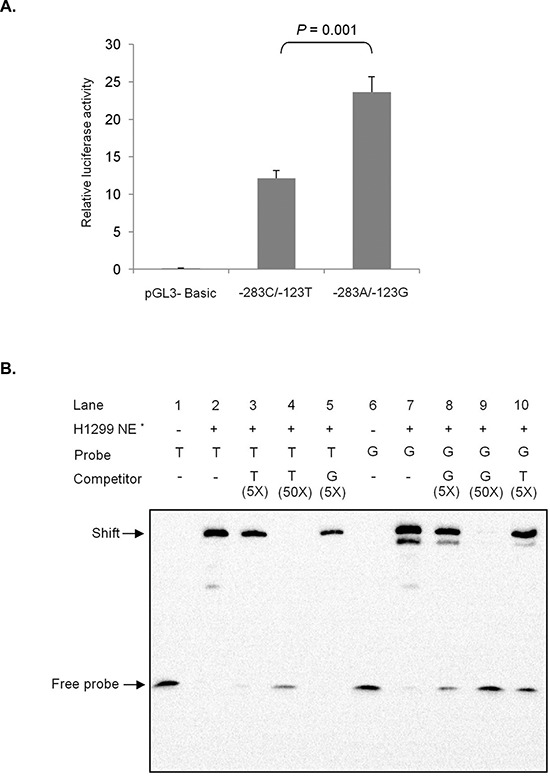
Effect of rs1279736C>A and rs3756585T>G (−283 and −123 from transcription start site, respectively) polymorphisms on RACK1 promoter **(A)** Transcription activity analysis of the haplotypes of rs1279736C>A and rs3756585T>G polymorphisms. The transcription activity of −283C/−123T haplotype and −283A/−123G haplotype was measured using Dual-Luciferase Reporter Assay System in H1299 cell line. The −283A/−123G haplotype had significantly increased promoter activity compared with the −283C/−123T haplotype. The results were confirmed in three independent experiments in triplicate. **(B)** Electrophoretic mobility shift assay with H1299 cell nuclear extracts using rs3756585T and rs3756585G oligonucleotides. Competition assays were performed using unlabeled rs3756585T or rs3756585G oligonucleotides. Each binding reaction contained 10 μg of nuclear extracts except lane 1 and 6, and labeled rs3756585T (lanes 1–5) or rs3756585G (lanes 6–10) oligonucleotides. Excess unlabeled rs3756585T (5- and 50-fold) and rs3756585G (5-fold) oligonucleotides were included in the binding reactions as competitors for labeled rs3756585T oligonucleotide (lanes 3–4 and 5, respectively). In addition, excess unlabeled rs3756585G (5- and 50-fold) and rs3756585T (5-fold) oligonucleotides were used to compete with rs3756585G oligonucleotide (lanes 8–9 and 10, respectively). *NE, nuclear extracts.

The effect of the haplotype of rs1279736C>A and rs3756585T>G polymorphisms on the promoter activity of the *RACK1* gene was investigated using a luciferase assay. In H1299 cells, the rs1279736A-rs3756585G haplotype significantly increased promoter activity compared to the rs1279736C-rs3756585T haplotype (*P* = 0.001, Figure 2A).

### The effect of *RACK1* rs3756585T>G on the binding activity of nuclear factors

Because the *RACK1* rs3756585T>G polymorphism is located at the promoter region of the *RACK1* gene, this SNP may affect *RACK1* transcription by modifying transcription factor binding. To test this hypothesis, we performed DNA-protein binding analysis using promoter fragments containing the SNP and nuclear extracts from H1299 cells. As shown in Figure 2B, the rs3756585G probe showed stronger nuclear protein binding than the rs3756585T probe. To verify the DNA-protein complex, competition assays were performed with specific and non-specific oligonucleotides. When the rs3756585G oligonucleotide was used to compete with the rs3756585T probe, it markedly disrupted the rs3756585T probe binding with nuclear protein. However, when a rs3756585T oligonucleotide was used to compete with the rs3756585G probe, it was not as effective as the rs3756585G oligonucleotide in disrupting nuclear protein binding. These results suggest that the rs3756585 T to G change increases transcription factor binding to the *RACK1* promoter, thereby increasing *RACK11* expression.

## DISCUSSION

We conducted a two-stage study using Affymetrix custom-made GeneChip to evaluate 1,385 SNPs in 910 candidate genes potentially involved in carcinogenesis to identify genetic variations associated with prognosis of patients with surgically resected early stage NSCLC. Of the 1,385 SNPs, five (*RACK1* rs1279736C>A and rs3756585T>G, *C3* rs2287845T>C, *PCAF* rs17006625A>G, and *PCM1* rs17691523C>G) were replicated across both stages of the study. In addition, this study provides evidence that the *RACK1* rs3756585 is a functional SNP. These findings suggest that five SNPs, particularly the *RACK1* rs3756585, could be used as prognostic markers for early stage NSCLC.

In this study, the rs1279736C>A and rs3756585T>G in the promoter of *RACK1* were most significantly associated with survival outcomes. *RACK1* is a cytosolic protein with seven internal Trp-Asp 40 (WD40) repeats and belongs to a WD40 family of proteins that includes the β subunit of G-proteins. *RACK1* was originally identified based on its ability to bind to the activated form of protein kinase C (PKC) isoform βII [7, 8]. As a scaffolding protein, RACK1 interacts with signaling molecules such as cyclic AMP-specific phosphodiesterase 4D isoform 5 (PDE4D5), the SRC family of tyrosine kinases, and β integrins, as well as PKC, and thus plays a pivotal role in a wide range of biologic responses, including cell growth, adhesion, and migration [8–12]. Several studies indicate that RACK1 plays an important role in cancer progression and that its expression is up-regulated during angiogenesis in several kinds of carcinomas, including lung cancer [13–16]. In addition, over-expression of *RACK1* has been reported to be strongly related to poor clinical outcomes of many carcinomas [14–17]. In the present study, *in vitro* promoter assay and EMSA revealed that the rs3756585 T-to-G change increased transcription factor binding and promoter activity of *RACK1*. Therefore, it is reasonable to expect that the *RACK1* rs3756585T>G lead to increased *RACK1* expression, thus contributing to poor survival outcomes.

In the present study, the *C3* rs2287845T>C (IVS22+7T>C) was also associated with survival outcomes. The complement system has a major role in innate and adaptive immunity. The *C3* protein is central to the activation of all the three complement pathways, the classical (in response to IgG- or IgM-antigen complexes), alternative (spontaneous activation), and mannose-binding lectin (in response to lectin residues on pathogen cell surface membrane) pathways [18, 19]. It has been reported that the complement system is activated in various types of cancer, including lung cancer [19–21]. Although complements have long been thought to function in immunosurveillance against tumors [19], there is growing evidence that complements play oncogenic roles in tumorigenesis [22–24]. Taken as a whole, these imply that the *C3* protein may contribute to the development and progression of lung cancer. Multiple alternatively spliced forms of the *C3* mRNA are present in human tissues (http://www.ensembl.org); A *C3* mRNA would produce truncated proteins that lack exon 1–34, another *C3* mRNA was expressed from exon 29 to exon 33b, and some of them produce non-coding RNAs. Although the functional significance of rs2287845T>C (IVS22+7T>C) remains to be elucidated, this splicing site polymorphism may lead to alternative splicing of *C3*, resulting in inter-individual variation in the expression levels of *C3* splicing variants. An alternative explanation is that the association between the rs2287845T>C and survival outcomes may be due to LD with other functional variants in the *C3* gene. As shown in (Supplementary Table 1), two of 7 potentially functional SNPs captured in the *C3* gene were evaluated in the discovery set because the remaining 5 SNPs could not be genotyped using the Affymetrix custom-made GeneChip. Therefore, further studies for these 5 SNPs and the causative functional SNP are needed in relation to survival outcomes.

PCAF, a member of the GCN5-related N-acetyltransferase (GNAT) family of protein acetyltransferases, was identified via its ability to interact with p300/CREB-binding protein (CBP) to form a multimeric acetylase complex [25, 26]. PCAF not only acetylates histones to facilitate gene transcription, but also acetylates some non-histone transcription factors, such as p53, to directly promote their transcription activity [27]. Evidence suggests that PCAF as a key regulator of these non-histone proteins can coordinate many carcinogenic processes, such as cell cycle progression, DNA damage response, and apoptosis [28–31]. PCM1, a ubiquitously expressed protein of 228 kDa with multiple coiled-coil domains, exhibits a distinct cell cycle–dependent association with the centrosome complex [32]. It has been shown that PCM1 plays an important role in the assembly of centrosomal proteins, microtubule organization, and cell cycle progression [33, 34]. There have been reports suggesting that *PCAF* and *PCM1* participate in the pathogenesis of several types of human malignancies [35–39]. The present study shows a reproducible association of *PCAF* rs17006625A>G and *PCM1* rs17691523C>G with survival outcomes. The PolyPhen algorithm [40] was used to predict functional relevance of *PCAF* rs17006625A>G, a non-synonymous SNP (Asn386Ser), and suggested that this change might be benign. The effect of rs17691523C>G on the promoter activity of *PCM1* was investigated using a luciferase assay, showing no difference between rs1769523C and rs1769523G alleles (data not shown). A possible explanation is that LD with other functional variants may be responsible for the effect of those two SNPs on survival outcomes. Further studies are needed to clarify the association between the SNPs and prognosis of patients with surgically resected NSCLC. In addition, further studies on the biologic function of those genes are needed to understand their roles in determining lung cancer prognosis.

In this study, the association between five SNPs and survival outcomes was replicated across both set of the study, which would largely reduce false positive findings in the genetic association study [23, 41]. Furthermore, our finding is biologically plausible in light of the putative function of the SNPs. However, several limitations in the present study should be considered. The modest sample size of both cohorts does not have optimal statistical power for discovering and validating the association, so some of the observed *P*-value did not reach to more stringent level of statistical significance that would avoid most of the false positive associations arising from multiple comparisons [41], limiting the reproducibility of the results. In addition, the sample size of the discovery set enables the identification of variants with a relatively large effect on survival outcomes, but does not have sufficient power for detecting variants with small effects on survival outcomes; therefore, there may be type II errors. Therefore, future studies with larger number of patients are required to validate our results. This study did not provide direct evidence that those five genes are involved in the development and progression of lung cancer, which limited our inquiry into the biologic mechanism of the observed associations between the SNPs and survival outcomes.

In conclusion, this study shows that five SNPs (*RACK1* rs1279736C>A and rs3756585T>G, *C3* rs2287845T>C, *PCM1* rs17691523, and *PCAF* rs17006625) influence survival outcomes of patients with surgically resected early stage NSCLC. Larger studies are required to confirm the effect of these SNPs in other ethnic populations.

## MATERIALS AND METHODS

### Study populations

A discovery set included 166 patients with pathologic stages I, II, or IIIA (micro-invasive N2) NSCLC who underwent curative surgical resection at the Kyungpook National University Hospital (KNUH), Daegu, Korea between September 1998 and December 2006. Genomic DNA samples from tumor and corresponding non-malignant lung tissue specimens were provided by the National Biobank of Korea at KNUH (Approval No. KNUHBIO_10–1016), which is supported by the Ministry of Health, Welfare and Family Affairs. Written informed consent was obtained from all patients prior to surgery. All materials derived from the National Biobank were obtained under Institutional Review Board approved protocols. For an independent validation set, a total of 626 patients were collected: 164 cases were obtained from the KNUH, 293 cases from Seoul National University Hospital, and 169 cases from Seoul National University Bundang Hospital. Written informed consent was obtained from all patients prior to surgery at each of the participating institutions and research protocol was approved by the institutional review boards at each institution. All of the patients included in this study were ethnic Koreans. None of the patients included in the discovery and validation sets received chemotherapy or radiotherapy prior to surgery. The pathologic staging of the tumors was determined according to the International System for Staging Lung Cancer [2].

### Selection of SNPs and genotyping

We selected SNPs for the present study using public database, as described previously [42]. Breifly, we selected 1,784 candidate genes involved in cancer-related pathways from the database of SABioscience (http://sabioscinece.com, Supplementary Table 1). To select all the potentially functional SNPs, we used the public database (http://www.ncbi.nlm.nih.gov/SNP). A total of 4,215 SNPs with minor allele frequency ≥ 5% in the HapMap JPT data were captured. Among those, 1,969 SNPs of 1,151 genes were genotyped using the Affymetrix custom-made GeneChip because other SNPs could not be applied to the platform. The lists of captured and genotyped SNPs in the discovery set are shown in Supplementary Table 1.

For validation, we selected and genotyped 56 SNPs which were associated with both overall survival (OS) and disease free survival (DFS) with *P_L-R_* < 0.05 under dominant, recessive, and/or codominant models in the discovery set using SEQUENOM's MassARRAY^®^ iPLEX assay (SEQUENOM Inc., San Diego, CA) or a restriction fragment length polymorphism assay.

### Promoter-luciferase constructs and luciferase assay

We investigated whether the rs1279736C>A and rs3756585T>G (−283 and −123 from transcription start site) modulate the promoter activity of receptor for activated C kinase 1 [*RACK1*] by luciferase assay. A 401bp fragment (from −378 to +21) including rs1279736C>A and rs3756585T>G was synthesized by PCR using genomic DNA from a donor carrying heterozygote at both SNPs. The forward primer with *Kpn*I restriction site (5′-GGGGTACCAATTAAGCTCCCCTGGGGTTG-3′) and the reverse primer with *HindIII* restriction site (5′-CCCAAGCTTCCGCCTTGCAGTGAAAGAGA-3′) were used. The PCR products were cloned into the *Kpn*I/*HindIII* site of the pGL3-basic plasmid (Promega, Madison, WI, USA). The correct sequence of all the clones was verified by DNA sequencing. The NLSCL cell line, H1299, was transfected with pRL-SV40 vector (Promega, Madison, WI, USA) and pGL3-basic plasmid using Effectene transfection reagent (Qiagen, Hilden, Germany). The cells were collected 48 h after transfection, and the cell lysates were prepared according to Promega's instruction manual. The luciferase activity was measured using a Lumat LB953 luminometer (EG & G Berthhold, Bad Wildbad, Germany), and the results were normalized using *Renilla* luciferase activity. All experiments were performed in triplicate.

### Electrophoretic mobility shift assay (EMSA)

EMSA was performed using the LightShift Chemiluminescent EMSA Kit (Pierce Biotechnology Inc, Rockford, IL, USA). Nuclear extracts were prepared from H1299 cells using Pierce NE-PER nuclear and cytoplasmic extraction reagents (Pierce Biotechnology Inc, Rockford, IL, USA). Ten micrograms of nuclear extracts were incubated with the probes at room temperature for 20 min. To identify DNA-protein binding specificity, competition assays were performed with 5- and 50-fold molar excesses of unlabeled competitor oligonucleotides prior to the addition of the labeled probe. The reaction mixture was resolved on a non-denaturing 6% acrylamide gel, and the gel was transferred to a positively charged nylon membrane. The membrane was UV cross-linked, and the biotin-labeled probe was detected using a stabilized Streptavidin-horseradish peroxidase conjugate.

### Statistical analysis

Differences in the distribution of genotypes according to the clinicopathologic factors of patients were compared using χ^2^ tests. OS was measured from the day of surgery until the date of death or to the date of the last follow-up. DFS was calculated from the day of surgery until recurrence or death from any cause. The survival estimates were calculated using the Kaplan-Meier method. The differences in OS and DFS across different genotypes were compared using the log-rank test. Hazard ratios (HRs) and 95% confidence intervals (CIs) were estimated using multivariate Cox proportional hazards models, with adjustment for age (≤ vs. > median age), gender (female vs. male), smoking status (never- vs. ever-smoker), tumor histology (Squamous vs. non-squamous), and pathologic stage (I vs. II-IIIA). A homogeneity test was performed to compare the difference between genotype-related HRs of different subgroups. All analyses were performed using Statistical Analysis System for Windows, version 9.2 (SAS Institute, Cary, NC, USA).

## SUPPLEMENTARY FIGURE AND TABLE




