# Supplementary material for: RACK1 is a candidate gene associated with the prognosis of patients with early stage non-small cell lung cancer
Source: Oncotarget. 2015 Jan 9;6(6):4451–66. doi: 10.18632/oncotarget.2865 (PMC4414203; doi:10.18632/oncotarget.2865)
Supplement: Supplementary file 1 [file oncotarget-06-4451-s001.pdf]

SUPPLEMENTARY FIGURE AND TABLE

A. ***RACK1*** **rs1279736C>A**

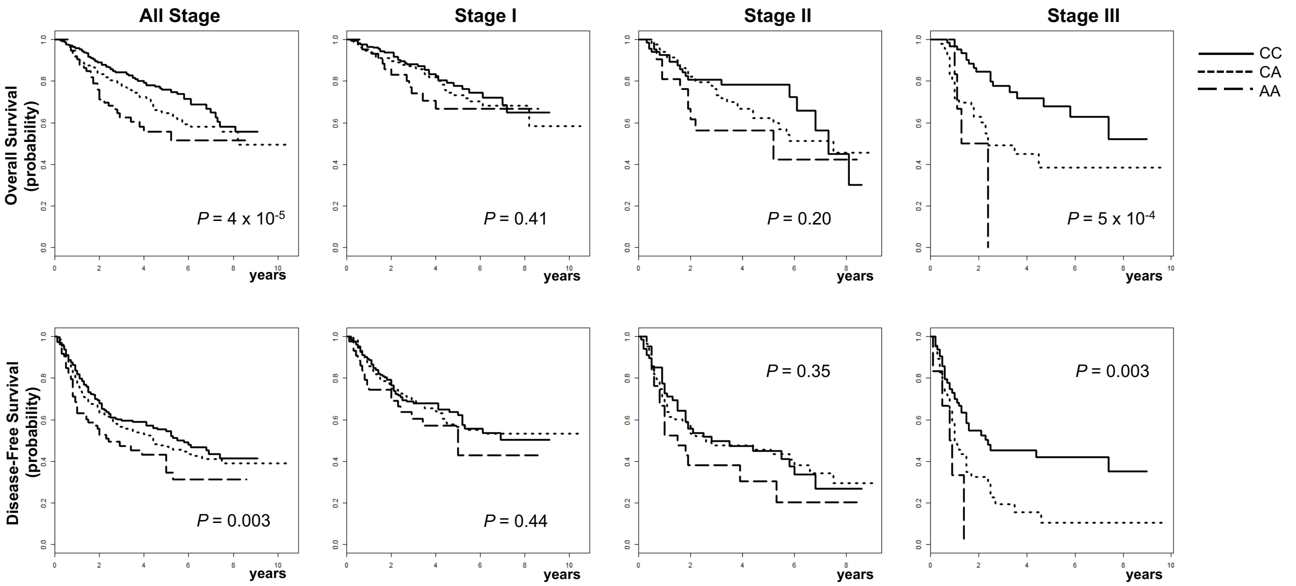

B. ***RACK1*** **rs3756585T>G**

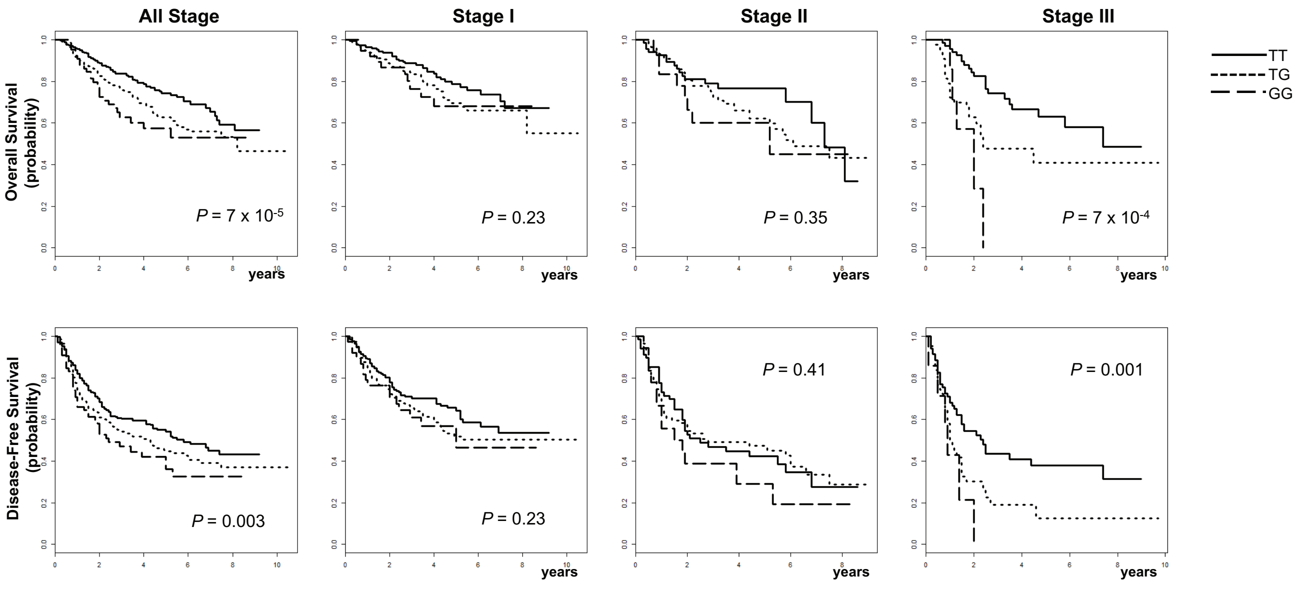

C. ***C3*** **rs2287845T>C**

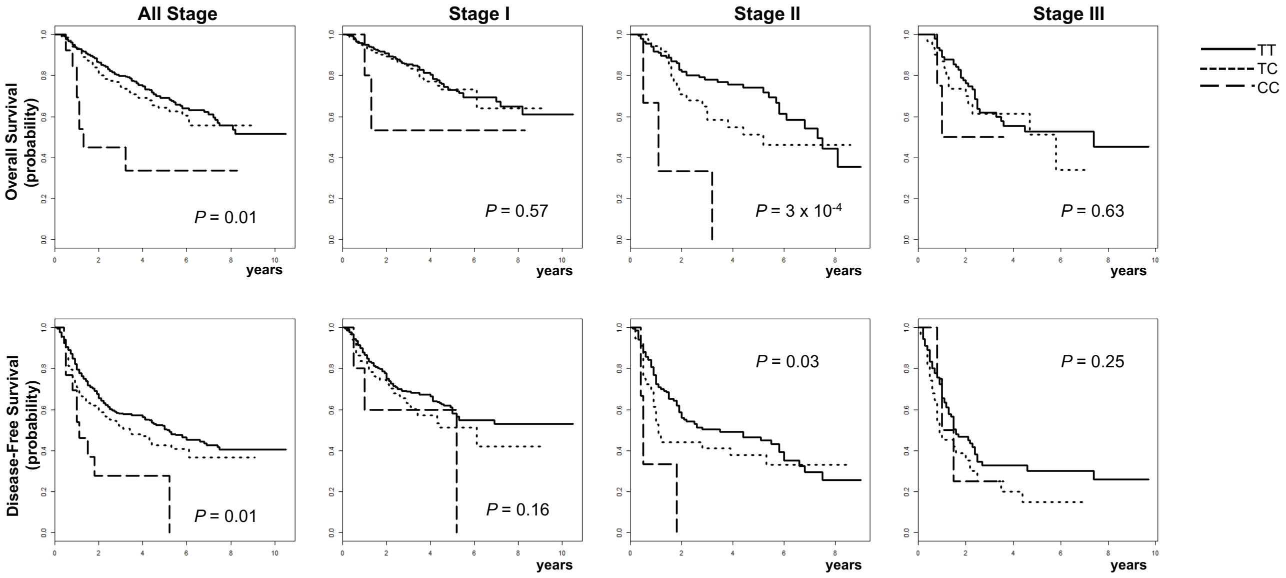

D. ***PCAF*** **rs17006625A>G**

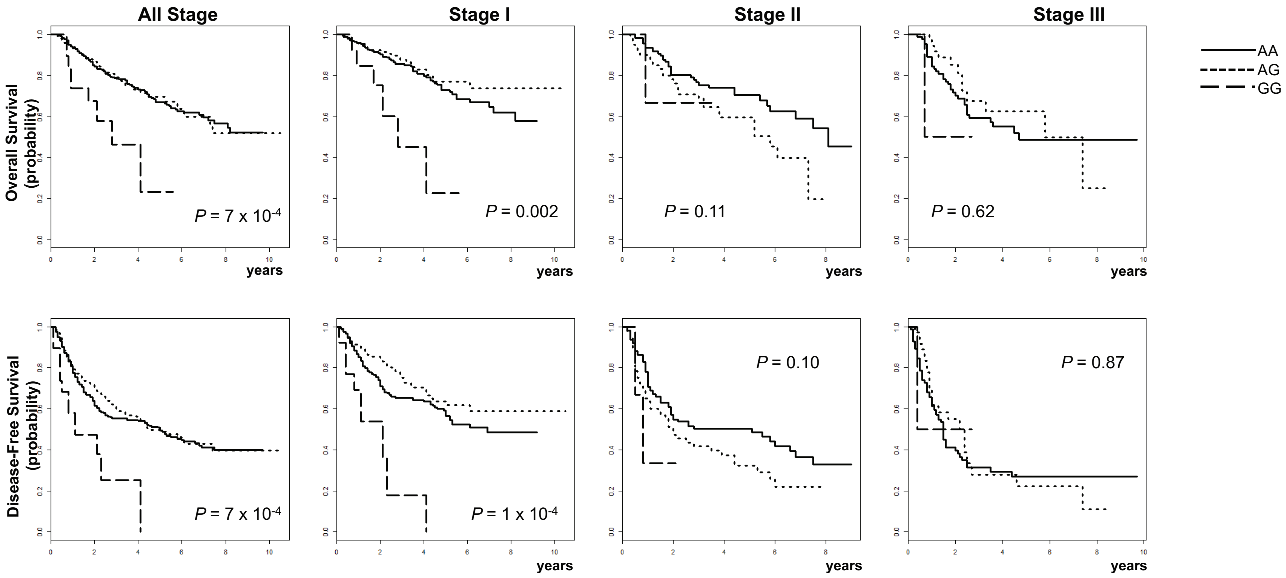

E. ***PCM1*** **rs17691523C>G**

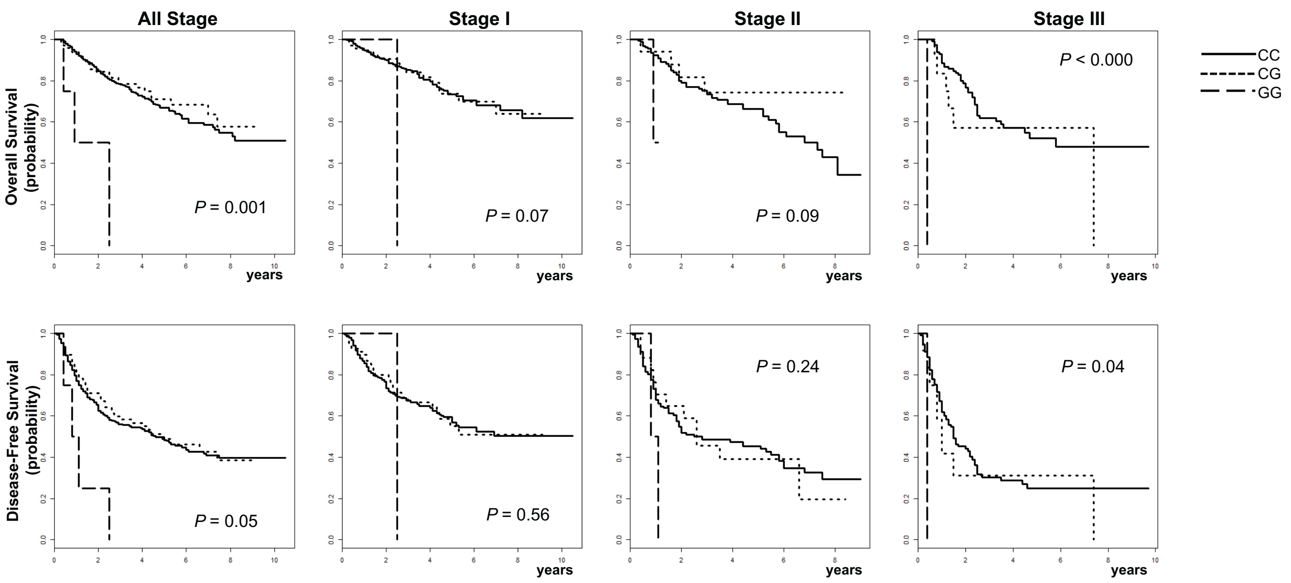

Supplementary Figure 1: Kaplan-Meier plots of overall survival and disease-free survival according to genotypes for all stages, stage I, stage II, and stage III in combined set of patients. *RACK1* rs1279736C > A, **(A)**; *RACK1* rs3756585T > G, **(B)**; *C3* rs2287845T > C, **(C)**; *PCAF* rs17006625A > G, **(D)**; and *PCM1* rs17691523C > G, **(E).**
